# Safety and efficacy of self-expandable metallic stent combined with ^125^I brachytherapy for the treatment of malignant obstructive jaundice

**DOI:** 10.1186/s40644-023-00551-0

**Published:** 2023-04-04

**Authors:** Ye Sheng, Xiaobo Fu, Guobao Wang, Maoyuan Mu, Weiwei Jiang, Zixiong Chen, Han Qi, Fei Gao

**Affiliations:** 1 Department of Minimally Invasive & Interventional Radiology, Sun Yat-sen University Cancer Center and Sun Yat-sen University State Key Laboratory of Oncology in South China, and Collaborative Innovation Center for Cancer Medicine, Guangzhou, Guangdong China; 2 Department of Interventional Radiology, The Third Affiliated Hospital of Soochow University and The First People’s Hospital of Changzhou, Changzhou, Jiangsu China; 3 Department of Endoscopy, Sun Yat-sen University Cancer Center and Sun Yat-sen University State Key Laboratory of Oncology in South China, and Collaborative Innovation Center for Cancer Medicine, Guangzhou, Guangdong China

**Keywords:** Malignant obstructive jaundice, Stent placement, Iodine-125 seed, Stent patency, Survival

## Abstract

**Background:**

Several previous studies demonstrated that the combination of self-expandable metallic stents (SEMS) and ^125^I seed implantation might prolong stent patency and obtain survival benefits for malignant obstructive jaundice (MOJ) patients. However, these studies rarely mentioned a comparison between CT-guided intratumoral ^125^I seed implantation and intraluminal ^125^I seed strand insertion combined with stenting for the management of MOJ. This study aimed to further evaluate the safety and efficacy of SEMS combined with ^125^I brachytherapy in the management of unresectable MOJ.

**Methods:**

Fifty-nine patients with unresectable MOJ were retrospectively included from March 2018 to June 2021. The main therapeutic outcomes were evaluated in terms of stent patency, and overall survival. Cumulative stent patency and overall survival rates were calculated by Kaplan–Meier survival analysis. Both clinical and treatment factors associated with survival were analyzed.

**Results:**

Technical success was achieved in all patients. The clinical success rate was 94% (32/34) in the seeds group and 92% (23/25) in the control group, no significant difference was found (*p* =1.000). The median duration of stent patency was significantly longer in the ^125^I brachytherapy group compared with the control group (289 days vs. 88 days, respectively, *p* =0.001). The ^125^I brachytherapy group demonstrated a significantly better median overall survival rate than the control group (221 days vs. 78 days, respectively, *p* =0.001). In multivariate analysis, stents with ^125^I brachytherapy (*p* =0.004) was a significant favorable prognostic factor that affected patient survival. No significant difference was observed between CT-guided ^125^I seed implantation and ^125^I seed strand insertion in stent patency (*p* =0.268), and overall survival (*p* =0.483).

**Conclusion:**

SEMS combined with ^125^I brachytherapy is safe and effective for treating MOJ. ^125^I brachytherapy may help to maintain stent patency and prolong overall survival. There was no significant difference between CT-guided ^125^I seed implantation with SEMS and ^125^I seed strand insertion with SEMS in stent patency and overall survival**.**

## Introduction

Malignant obstructive jaundice (MOJ) is one of the most prevalent complications resulting from biliary invasion or compression by various malignancies, such as cholangiocarcinoma, pancreatic carcinoma, gallbladder carcinoma, and liver cancer [1–3]. Although surgical resection remains the gold-standard radical therapy for MOJ, more than 80% of patients are detected in an unresectable stage due to its silent and insidious clinical processes [4, 5]. Biliary stent deployment has been widely accepted as the preferred palliative modality for inoperable MOJ patients. It can effectively relieve symptoms of jaundice, improve quality of life, and prolong survival [6, 7]. Nevertheless, stent restenosis occurred in more than half of patients within six months after implantation. Tumor ingrowth and epithelial cell proliferation may contribute to stent dysfunction [8]. Since a self-expandable metallic stent (SEMS) itself has no therapeutic effect on tumors, additional efforts are required to maintain stent patency and improve patients’ survival.

Recent years have witnessed a growing academic interest in ^125^I brachytherapy, which has been proven to be beneficial in various malignancies [9–11]. Previous studies have concluded that the combination of SEMS and ^125^I seed implantation might prolong stent patency and even improve survival for MOJ patients [12–14]. However, the ^125^I brachytherapy techniques differed in these studies, and no study has reported a comparison between CT-guided intratumoral ^125^I seed implantation and intraluminal ^125^I seed strand insertion combined with stenting for the management of MOJ. Therefore, this retrospective research aimed to further assess the safety and effectiveness of SEMS combined with intratumoral or intraluminal ^125^I brachytherapy in the management of unresectable MOJ, and a subgroup analysis of the two approaches of deployment ^125^I was conducted.

## Materials and methods

### Patients

This retrospective cohort study conformed to the Declaration of Helsinki of the World Medical Association (2013) and was approved by the Institutional Review Board of our center that waived the need for written informed consent. A comprehensive search was conducted using our database between March 1, 2018, to June 31, 2021. One hundred and thirty-seven patients were diagnosed with malignant biliary obstruction.

Inclusion criteria were: (1) a confirmed unresectable malignant biliary obstruction diagnosis evaluated by two senior surgeons based on laboratory, pathological, or radiologic evidence; (2) the presence of obstructive jaundice; (3) patients who received stent placement alone or coupled with ^125^I brachytherapy. (4). Life expectancy > 3 months.

Exclusion criteria: (1) received previous biliary interventional treatment before admission (2) received endoscopic or percutaneous biliary drainage without stent placement. (3) Incomplete medical record; (4) Lost follow-up.

**Fig. 1:**
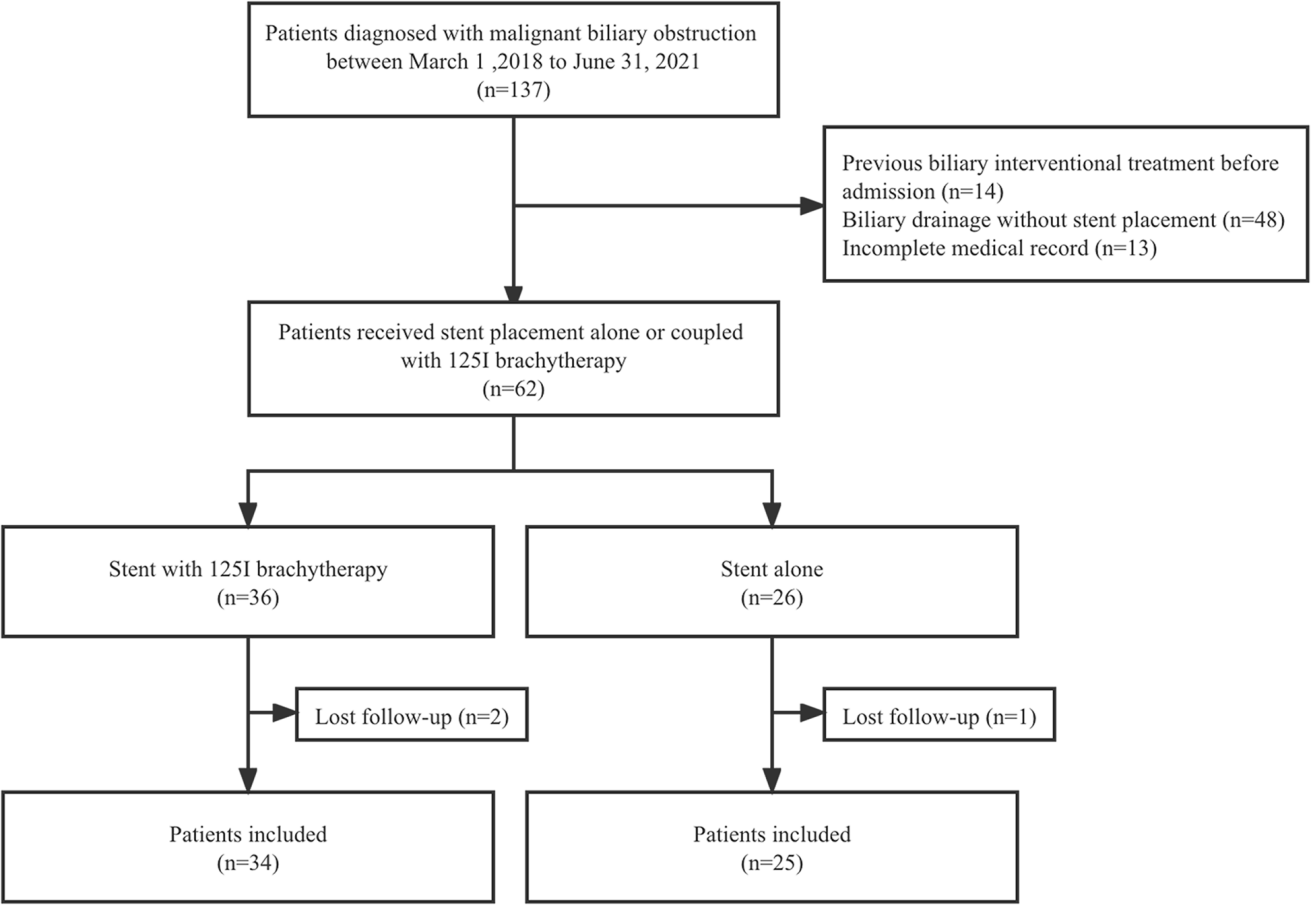
Patient flow diagram

Seventy-eight patients were eliminated from the study: received previous biliary interventional treatment before admission(*n* =14); patients received endoscopic or percutaneous biliary drainage without stent placement(*n* =48); incomplete medical record(*n* =13); lost follow-up(*n* =3). Among the 59 patients included, 34 patients chose the combined therapy (^125^I brachytherapy group) including 20 patients underwent stent placement with intraluminal ^125^I seed strand insertion, and 14 underwent stent placement with intratumoral ^125^I seed implantation under CT-guidance. Twenty-five patients were treated with the biliary stent alone (the control group) (Fig. 1).

### Outcomes and definitions

The endpoints in this study were technical success, clinical success, stent patency, patient survival, and complications. Technical success was classified as satisfactory placement of the stent through the stricture with the seed strand or seeds in the appropriate position and brisk flow of contrast through the stent into the intestine. Clinical success was defined as the serum total bilirubin level dropped by 75% of the preoperative value within 4 weeks [15]. The duration of stent patency was defined as the interval between stent deployment and stent restenosis. The definition of stent restenosis was a recurrence of cholangitis or jaundice, a rise in bilirubin level, and biliary dilation verified by imaging examination or cholangiography. If a patient died without stent restenosis, the patency duration was regarded as equivalent to the survival period but censored. Survival was measured from the date of preliminary stent deployment to death or the end of the study. According to the American Society of Interventional Radiology’s recommendations, complications were typically categorized as major or minor [16]. Major complications referred to those that result in death or serious adverse effects including sepsis, uncontrollable biliary or intestinal bleeding, and acute renal failure; others were considered minor complications.

### Follow-up

All patients were followed up regularly until December 2021 or patient death. The follow-up visits consisted of routine outpatient and telephone interviews. Outpatient interviews were conducted 1 month after stent implantation and every 2 months thereafter. Telephone interviews were performed every 3 months after stent implantation. For patients with stent restenosis confirmed by imaging examination or cholangiography, stent revision or external biliary drainage was considered.

### ^125^I seeds and stents

The ^125^Iseed (Larvin Bioengineering Technology Company) consists of a core radioactive source and a titanium capsule. The outer diameter of the iodine-125 seeds was 0.08 mm, and the length of the seeds was 4.45 mm. The measured emissions were 27–32 keV X-rays and 35.5 keV γ-rays with a half-life of 59.6 days. Each seed had an effective radiation radius of 1.7 cm and radioactivity of 0.8 mCi. The uncovered SEMS (Wallstent [Boston Scientific, Natick]) selected in this study had a diameter of 8 mm with lengths ranging from 40 to 80 mm.

### Preparation before procedure

A comprehensive set of biochemical and hematological examinations were performed before and after the procedure. Contrast-enhanced computed tomography (CT) or magnetic resonance imaging (MRI) scans were conducted to confirm the location of the biliary stricture and the extent of intrahepatic bile duct dilatation.

### ^125^I brachytherapy group

**Fig. 2:**
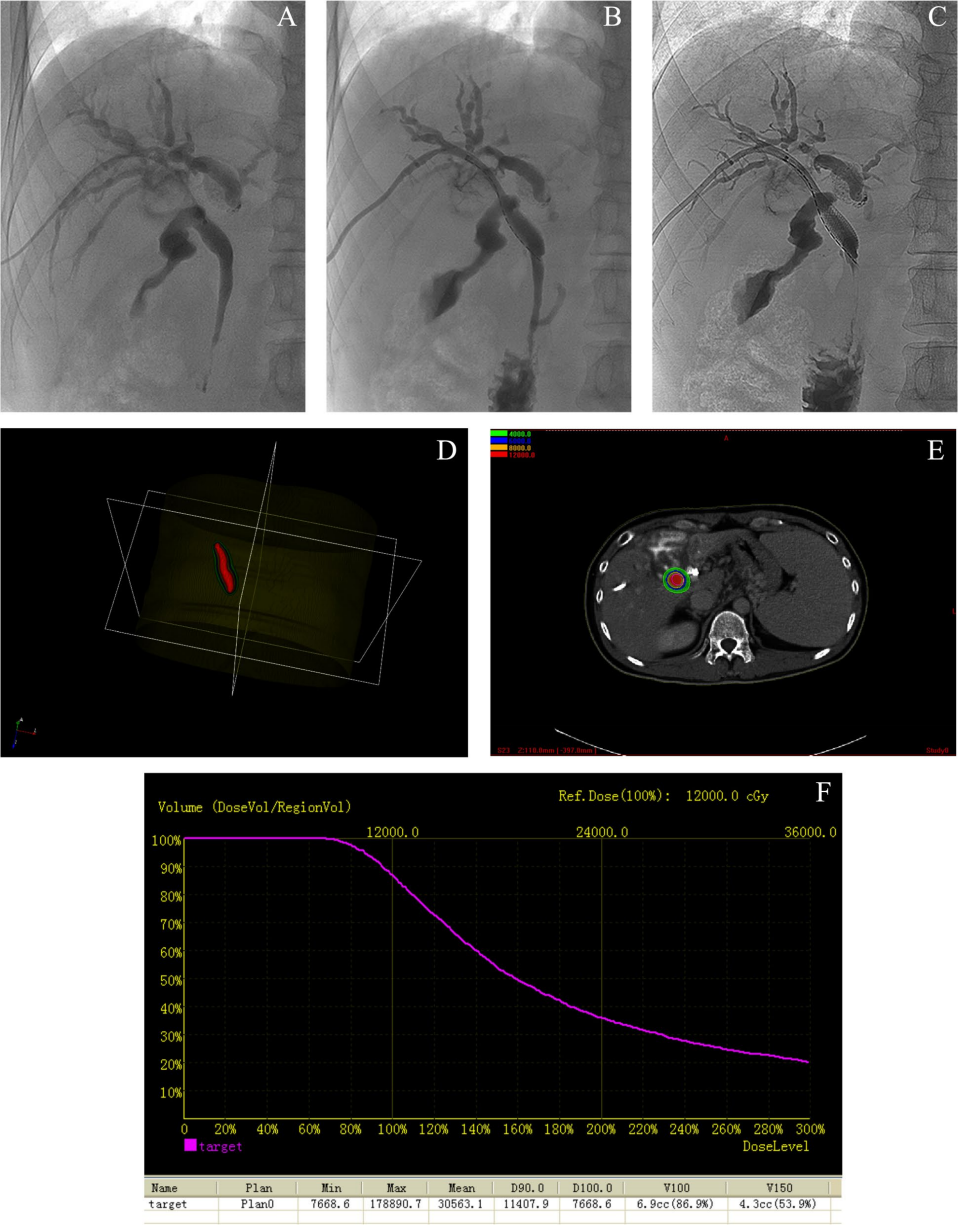
A 47-year-old male patient with hepatocellular carcinoma received SEMS insertion plus ^125^I seed strand insertion. **A** Percutaneous transhepatic cholangiography showed stenosis of the common hepatic duct. **B** Cholangiography showed the process of insertion of ^125^I seed strand (containing 15 seeds). **C** Cholangiography showed an 8×60 mm self-expandable metallic stent was placed accurately into the biliary stricture. (Arranged ^125^I seed strand between the stent and biliary wall). **D** Three-dimensional reconstructed image of ^125^I seed strand. **E** Dose distribution of ^125^I seed strand on TPS. **F** Dose-volume histogram calculated after ^125^I seed strand insertion

The number of ^125^I seeds to be implanted was determined according to the plan of the preoperative computerized treatment planning system (TPS) (RT-RSI, Beijing Atom, and High Technique Industries Inc.). A 4F catheter was used to linearly arrange the seeds. Both ends of the catheter were clipped to match the length of the seed string and to prevent leakage. A heated vascular clamp was used to seal the catheter’s two ends. The stent and seed strand implantation techniques were conducted under fluoroscopic supervision. A 22-G Chiba needle (Cook, Bloomington, IN) was used to percutaneously puncture the dilated peripheral intrahepatic bile duct. Then, a Neff percutaneous access set was introduced into the bile duct, and cholangiography was performed through the outer cannula to assess the location and length of the stenotic site. A 0.035-inch, 180-cm long guidewire was advanced to cross the blockage location, and the outer cannula was introduced over the guidewire to cross the stenosis. Subsequently, the guidewire was withdrawn, and the seed strand was delivered through the outer cannula to the target position. After seed strand implantation, the outer cannula was removed, and the stiff guidewire was inserted again into the distal duodenum. A bare SEMS (Wallstent [Boston Scientific, Natick]) of appropriate size was introduced and placed in the middle of the stenosis. The stent stretched at least 2 cm beyond the stenosis on each side, and the seed strand was attached consistently between the stent and biliary wall. Repeat cholangiography was conducted to confirm stent patency and location of the seed strand. Finally, CT was scheduled 1 week after the procedure, with the images imported into the TPS to assess the dosage and distribution of the ^125^I seed strand (Fig. 2).

**Fig. 3:**
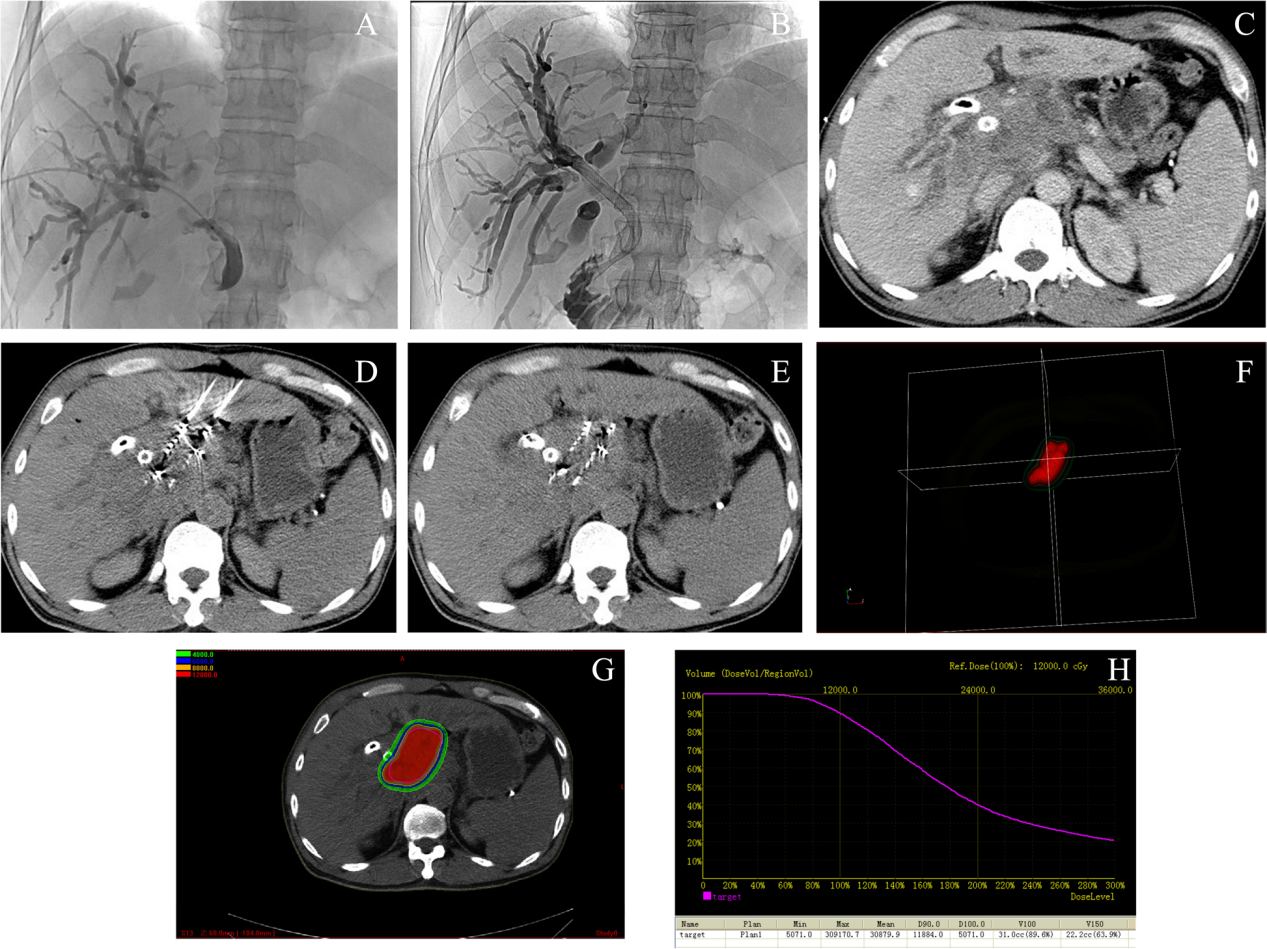
A 47-year-old male patient with cholangiocarcinoma received SEMS insertion plus CT-guided percutaneous ^125^I seed implantation. **A** Cholangiography showed the obstruction of the common bile duct. **B** Cholangiography showed an 8×80 mm self-expandable metallic biliary stent insertion. **C** Computed tomography showed the mass of malignancy. **D** Computed tomography showed the image of the percutaneous ^125^I seed implantation process. **E** Computed tomography showed the seeds’ distribution after ^125^I seed implantation. **F** Three-dimensional reconstructed image of the implanted seeds. **G** Dose distribution of the seeds on TPS. **H** Dose-volume histogram calculated after CT-guided percutaneous ^125^I seed implantation

For patients who underwent stent placement with ^125^I seed implantation under CT guidance, the technique for placing the stent was similar to that mentioned before. After stent placement, CT-guided ^125^I seed implantation was performed. The puncture approach was displayed on the immediate CT scan image according to the computerized TPS. It was necessary to penetrate the target lesion with an 18 G seed needle (Larvin Bioengineering Technology Company) under CT guidance to ensure the precision of the depth and angle of the needle. Subsequently, the needle core was removed, and seeds were released through the outer cannula into the lesion by using a seed implantation gun (Larvin Bioengineering Technology Company). Each seed was released while drawing back the needle every 0.5 cm. A final CT scan was performed to validate the implanted seeds’ distribution and confirm the lack of postoperative complications. The dosage and distribution of the implanted seeds were assessed according to TPS after brachytherapy (Fig. 3).

### Control group

The stent placement process in the control group was nearly identical to that in the ^125^I brachytherapy group. After stent implantation, repeat cholangiography was obtained to assess stent position and patency.

### Statistical analysis

Continuous variables were expressed as the mean ± standard deviation and analyzed by the independent t test or paired t test. Categorical variables were expressed as frequency and percentage, and compared by the chi-squared test or Fisher’s exact test. Cumulative stent patency and overall survival rates were calculated by Kaplan–Meier survival analysis. The log-rank test (Mantel-Cox) was performed to compare survival among different groups. To confirm the independent prognostic factors that affected patient survival, a Cox regression model was built for the univariate and multivariate analyses. All variables with *p* <0.100 in univariate analyses were included in the subsequent multivariate analysis. Statistical significance was considered only when a *p*-Value was less than 0.05. All statistical analyses were performed by using IBM SPSS for Windows version 26.0 (SPSS, Chicago, Illinois, USA).

## Results

### Patient characteristics

A total of 59 patients (39 men and 20 women) with a mean age of 58.7 years (range 28-86 years) were included. The etiologies included cholangiocarcinoma (*n* = 22), hepatocellular carcinoma (*n* = 14), and metastases associated with different malignancies (*n* = 23). Twenty-three patients had their diagnoses established by pathologic examination, while the remaining 36 patients had their diagnoses determined by radiological and laboratory tests. The obstruction site was the hilar biliary tract in 52 individuals and the intermediate and distal biliary tract in 7 individuals. Fifteen patients received sequential systemic anti-cancer therapy. There was no statistical significance in the characteristics between the two groups (all *p* >0.05). Patients’ baseline characteristics are presented in Table 1.

**Table 1:** Patient characteristics

Characteristics	All (*n*=59)	Brachytherapy group (*n*=34)	Control group (*n*=25)	*P* value
Age (year)	58.71±13.26	60.79±12.56	55.88±13.90	0.161
Sex (male/female)				0.396
male	39 (66.1%)	24 (70.6%)	15 (60.0%)	
female	20 (33.9%)	10 (29.4%)	10 (40.0%)	
Location of obstruction				0.687
Hilar bile duct	52 (88.1%)	29 (85.3%)	23 (92.0%)	
Middle and distal bile duct	7 (11.9%)	5 (14.7%)	2 (8.0%)	
Etiology				0.063
HCC	14 (23.7%)	9 (26.5%)	5 (20.0%)	
Cholangiocarcinoma	22 (37.3%)	16 (47.1%)	6 (24.0%)	
Metastatic cancer	23 (39.0%)	9 (26.4%)	14 (56.0%)	
Bismuth type				0.149
II	5 (8.5%)	4 (11.8%)	1 (4%)	
III	7 (11.9%)	6 (17.6%)	1 (4%)	
IV	47 (79.6%)	24 (70.6%)	23 (92%)	
ECOG				0.643
1	42 (71.2%)	25 (73.5%)	17 (68.0%)	
2	17 (28.8%)	9 (26.5%)	8 (32.0%)	
Obstruction length (cm)	3.24±0.69	3.22±0.69	3.26±0.70	0.817
Stent number				0.166
one	10 (16.9%)	8 (23.5%)	2 (8.0%)	
two	49 (83.1%)	26 (76.5%)	23 (92.0%)	
Systemic anti-tumor therapy				0.154
Yes	15 (25.4%)	11 (32.4%)	4 (16.0%)	
No	44 (74.6%)	23 (67.6%)	21 (84%)	

*HCC* Hepatocellular carcinoma, *ECOG* Eastern Cooperative Oncology Group

### Technical success

Technical success was achieved in every patient in both groups. A total of 108 stents were implanted in 59 patients, of which 8 (23.5%) in the brachytherapy group and 2 (8.0%) in the control group received one stent, while 26 (76.5%) in the brachytherapy group, and 23 (92.0%) in the control group received two stents (*p* =0.166). Stents measuring 8×80 mm and 8×60 mm were most frequently used. Approximately 27.6 seeds (range 12-40) were implanted as a strand on average, while the mean number of seeds implanted under CT guidance was 36.1 (range 16-77). None of the seeds were lost during the delivery and deployment process.

### Clinical success

The clinical success rate was 94% (32/34) in the brachytherapy group and 92% (23/25) in the control group (*p* =1.000). Among the four individuals who failed to achieve clinical success, one in the brachytherapy group who developed liver abscess received anti-infective therapy, two with post-procedure cholangitis (one in the brachytherapy group and the other one in the control group) were treated medically, and the other patient in the control group underwent reintervention because of stent restenosis. Statistically significant improvements were observed from before to one month after the procedure in liver function values, including total bilirubin (TBIL), direct bilirubin (DBIL), alanine aminotransferase (ALT), and aspartate aminotransferase (AST) (all *p* <0.05) (Table 2).

**Table 2:** Liver function parameters before and 1 month after the procedure

Parameters	All (*n*=59)	Brachytherapy group (*n*=34)	Control group (*n*=25)	*P* value
ALT (U/L)				
Before	57.58±26.66	60.51±25.58	53.60±28.09	0.329
After	44.71±16.64	46.97±16.36	41.62±16.85	0.226
*P* value		0.002*	0.004*	
AST (U/L)				
Before	68.87±33.10	65.57±30.74	73.35±36.22	0.377
After	46.66±19.84	45.59±21.22	48.10±18.13	0.635
*P* value		0.000*	0.000*	
TBIL (μmol/L)				
Before	324.47±186.75	305.18±194.12	350.71±176.69	0.359
After	65.07±31.09	61.93±33.48	69.34±27.60	0.370
*P* value		0.000*	0.000*	
DBIL (μmol/L)				
Before	260.93±142.22	247.43±152.21	279.29±128.12	0.400
After	54.19±27.41	51.02±29.38	58.50±24.40	0.304
*P* value		0.000*	0.000*	

Liver function parameters including ALT, AST, TBIL, and DBIL decreased significantly from before to 1 month after the procedure, * p value ≤ 0.05 was considered to indicate statistical significance

*ALT* Alanine aminotransferase, *AST* Aspartate aminotransferase, *TBIL* Total bilirubin, *DBIL* direct bilirubin

### Complications

No procedure-related major complications were observed in the ^125^I brachytherapy group and the control group. Minor complications occurred in 12 patients (20.3%) in the two groups, including three patients (5%) who experienced cholangitis, and one patient (1.7%) in the ^125^I brachytherapy group experienced liver abscess; these infections were well controlled with antibiotics. Five patients (8.5%) in the two groups suffered severe abdominal pain, which was relieved after using analgesics. Two patients (3.4%) in the ^125^I brachytherapy group with self-limited hemobilia were treated with conservative management. In addition, seed strand migration in the ^125^I brachytherapy developed in one patient (1.7%). Considering that the dislocated seed strand still covered most of the stenosis site, a reintervention procedure was not performed. There was no significant difference in related complications between the two groups (all *p* >0.05) (Table 3).

**Table 3:** complications related to the procedure

Complications	All (*n*=59)	Brachytherapy group(*n*=34)	Control group(*n*=25)	*P* value
Cholangitis	3 (5.1%)	2 (5.9%)	1 (4%)	1.000
Pancreatitis	0 (0%)	0 (0%)	0 (0%)	-
Self-limited hemobilia	2 (3.4%)	2 (5.9%)	0 (0%)	0.503
Stent or seeds migration	1 (1.7%)	1 (2.9%)	0 (0%)	1.000
Severe abdominal pain	5 (8.5%)	3 (8.8%)	2 (8%)	1.000
Liver abscess	1 (1.7%)	1 (2.9%)	0 (0%)	1.000

### Stent patency and overall survival

**Fig. 4:**
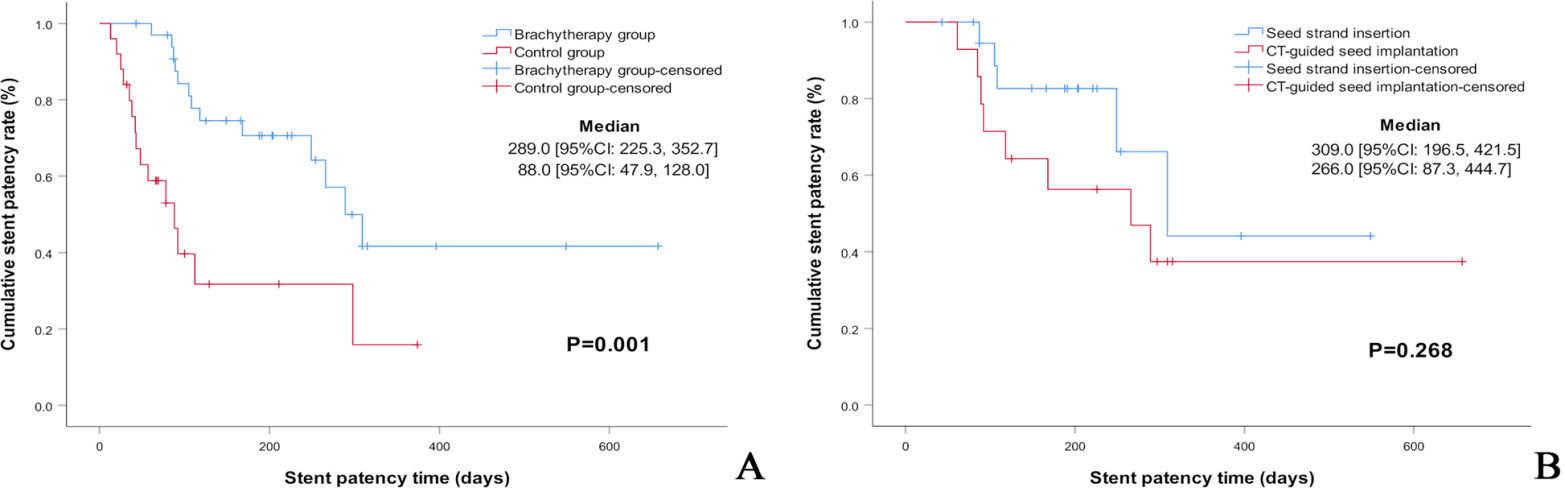
Kaplan–Meier analysis of stent patency. **A** Comparison of the stent patency of the ^125^I brachytherapy and the control group. **B** Comparison of the stent patency of CT-guided ^125^I seed implantation and ^125^I seed strand insertion. (Subgroup analysis)

**Fig. 5:**
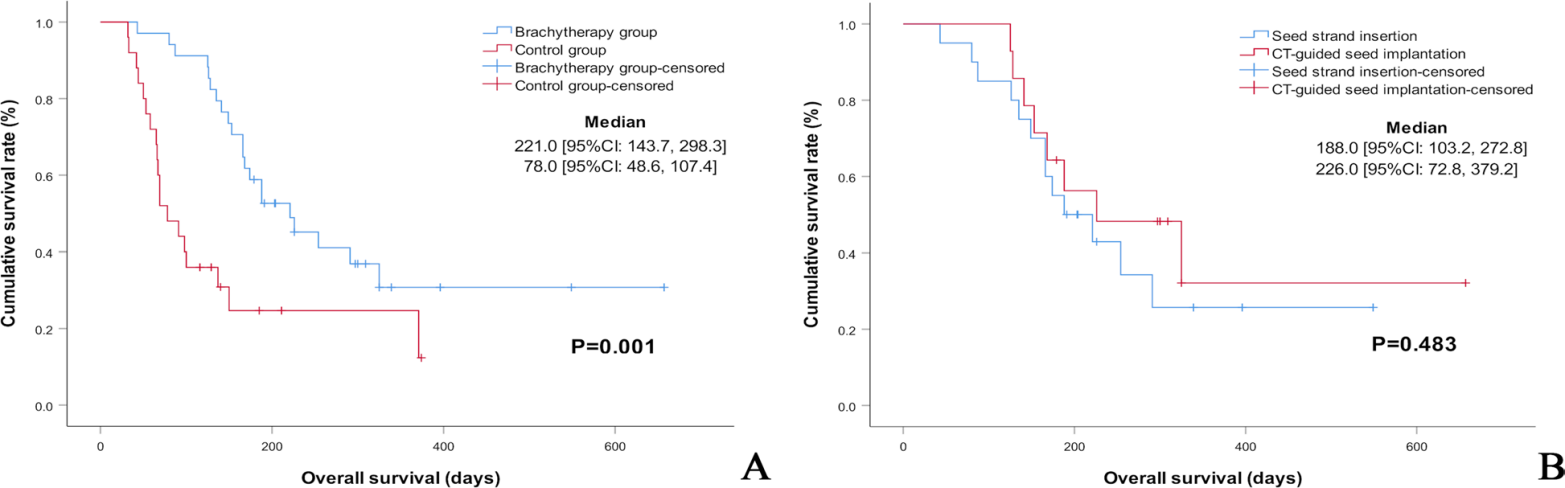
Kaplan–Meier analysis of patients’ survival. **A** Comparison of the overall survival of the ^125^I brachytherapy group and the control group. **B** Comparison of the overall survival of CT-guided ^125^I seed implantation and ^125^I seed strand insertion. (Subgroup analysis)

The ^125^I brachytherapy group had a significantly longer median duration of primary stent patency than the control group (289 days, 95% CI: 225.3 - 352.7 days vs. 88 days, 95% CI: 47.9 - 128.0 days, respectively, *p* =0.001) (Fig. 4A). The median overall survival in the ^125^I brachytherapy group was considerably superior to the control group (221 days, 95% CI: 143.7 - 298.3 days vs. 78 days, 95% CI: 48.6-107.4 days, respectively, *p* =0.001) (Fig. 5A).

In subgroup analysis, there was no significant difference in the median duration of primary stent patency between CT-guided ^125^I seed implantation and ^125^I seed strand insertion (266 days, 95% CI: 87.3 - 444.7 days vs. 309 days, 95% CI: 196.5 - 421.5 days, respectively, *p* =0.268) (Fig. 4B). No significant difference in median overall survival was observed between CT-guided ^125^I seed implantation and ^125^I seed strand insertion ((226 days, 95% CI: 72.8 - 379.2 days vs. 188 days, 95% CI: 103.2 - 272.8 days, respectively, *p* =0.483) (Fig. 5B).

### Prognostic factors

As shown in Table 4, in a univariate analysis to determine the prognostic variables affecting the survival of included patients, Systemic cancer treatment, and stent with brachytherapy were statistically significant in univariate analysis (*p* <0.100). Ultimately, two items were included in the multivariate analysis. and stent with brachytherapy ([Hazard Ratio (HR)]:0.383, *p* =0.004),) was a favorable prognostic factor.

**Table 4:** Multivariate analyses of variables that affected the survival of included patients(*N*=59)

Variables	Univariate analysis			Multivariate analysis		
	Hazard ratio	95% CI	*P* value	Hazard ratio	95% CI	*P* value
Age	0.995	0.971-1.020	0.703			
Gender						
Male	1					
Female	0.688	0.349-1.355	0.280			
Location of obstruction						
Hilar bile duct	1					
Middle and distal bile duct	0.947	0.394-2.274	0.902			
Etiology						
HCC	1					
Cholangiocarcinoma	0.621	0.283-1.365	0.236			
Metastatic cancer	0.759	0.343-1.680	0.497			
Bismuth type						
II	1					
III	0.783	0.157-3.890	0.765			
IV	1.652	0.505-5.404	0.407			
ECOG						
1	1					
2	0.839	0.408-1.725	0.634			
Obstruction length	1.359	0.867-2.131	0.181			
Stent number						
one	1					
two	0.763	0.336-1.734	0.518			
Systemic anti-tumor therapy						
No	1			1		
Yes	0.493	0.218-1.116	0.090*	0.580	0.254-1.327	0.197
Treatment						
Stent alone	1			1		
Stent with brachytherapy	0.354	0.186-0.673	0.002*	0.383	1.365-5.005	0.004*

*HCC* Hepatocellular carcinoma, *ECOG* Eastern Cooperative Oncology Group

^*^p value ≤ 0.100 was considered to indicate statistical significance

## Discussion

Currently, few data are available comparing CT-guided intratumoral ^125^I seed implantation and intraluminal ^125^I seed strand insertion combined with stents for the treatment of MOJ [12–14, 17]. Therefore, this research compared the clinical outcomes of patients who had stents insertion with seed strand placement or CT-guided ^125^I seed implantation to those who had stents insertion alone. The results showed that SEMS combined with ^125^I brachytherapy may help to maintain stent patency and prolong overall survival. Moreover, no significant difference was found between CT-guided ^125^I seed implantation with SEMS and ^125^I seed strand insertion with SEMS in stent patency and overall survival**.**

Generally, the placement of uncovered metallic stents can rapidly reduce jaundice and improve patients’ quality of life. SEMS has been recognized as one of the primary treatments for MOJ cases. Unfortunately, stent restenosis owing to invasive tumor growth, inflammation, and tissue hyperplasia has been reported as a nonnegligible limitation of uncovered SEMS [18, 19]. Although covered stents are designed to avoid the above problem, higher stent migration rates were reported compared to uncovered stents. Moreover, the covering membrane may block collateral bile ducts, resulting in acute cholecystitis, pancreatitis, and other complications [20–22]. Therefore, an efficient technique to not only inhibit tumor ingrowth physically like covered stents but also induce tumor regression is highly desired.

A series of clinical investigations have demonstrated that ^125^I brachytherapy is effective in treating malignant tumors [9–11]. Compared with traditional external radiotherapy, ^125^I seed brachytherapy can deliver a higher effective dose of radiation into the tumor with a limited influence on surrounding normal tissues and adjacent organs. Additionally, ^125^I seeds can continuously release X- and γ-rays onto the tumor cells, efficiently decreasing cell proliferation and increasing cell sensitivity to radiation [23]. Furthermore, as a form of conformal radiotherapy, ^125^I brachytherapy is associated with minimal treatment errors owing to patient mobility.

Chen et al. [24] showed that intraluminal brachytherapy using an ^125^I seed strand fixed in a drainage catheter through a stent was a safe and effective therapy for MOJ patients, but no conclusions regarding patient survival were reported. Guo et al. [25] first reported the use of a SEMS loaded with ^125^I seeds to treat advanced esophageal cancer. Zhu et al. [10] studied the application of irradiation biliary stents loaded with ^125^I seeds in the treatment of malignant biliary obstruction. It demonstrated that ^125^I seed-loaded biliary stents significantly prolonged stent patency and patients’ survival compared to the control group. Several more recent clinical studies on the combination of ^125^I seeds and SEMS have been attempted, which also demonstrated that the combined technique can offer significantly better stent patency and patients’ survival than stent alone [11, 14, 26, 27]. The current study demonstrated that the median duration of primary stent patency was significantly longer in the brachytherapy group compared with the control group (289 vs. 88 days, respectively, *p* =0.001). In this study, the median overall survival was considerably better in the brachytherapy group than in the control group (221 vs. 78 days, respectively, *p* =0.001). This study also confirmed that the combination of SEMS with ^125^I brachytherapy was a favorable prognostic factor associated with patient survival. Meanwhile, the TBIL and DBIL dropped dramatically one month after the procedure in both groups. The above results seemingly suggest that the ^125^I brachytherapy group had longer stent patency and patient survival. It demonstrated that additive ^125^I brachytherapy would considerably alleviate compression or invasion of tumors in patients.

However, in subgroup analysis, no significant difference between CT-guided ^125^I seed implantation and ^125^I seed strand insertion was found in stent patency and median overall survival (all *p* >0.05). The small sample size and difference in patient selection might explain part of this result. In the present study, we found that both two ^125^I brachytherapy techniques were safe and effective in treating MOJ, and the choice of technique depended on the nature and location of the tumor. The ^125^I seed strand insertion was preferred for the invasive growth tumor along the bile duct wall, especially when the common bile duct was invaded. As most such tumors had short diameters, implanting seeds directly into the tumor was difficult. While the combined therapy of SEMS with ^125^I seed strand insertion presented various advantages for such advanced tumors invading the bile duct wall. First, the delivery of the ^125^I seed strand through the outer cannula could reduce the potentially adverse events related to multiple direct puncturing of the tumor for radioactive seed implantation. Second, because the seeds were linearly sealed into a catheter to construct a seed strand and fixed steadily between the stent and biliary wall, the seed dislodgment probability after implantation could be decreased. Third, the ^125^I seed strand was accurately attached in the diseased section by the force of SEMS, which can effectively inhibit tumor cell invasion and epithelial cell hyperplasia around the bile duct wall, thereby delaying the recurrence of stent stenosis. On the other hand, for tumors that invaded the right and left intrahepatic bile duct or distal from the bile duct, CT-guided ^125^I seed implantation was considered to be the first choice. First, due to the short radiation radius of the ^125^I seeds, the intraluminal seed strand is difficult to thoroughly eliminate the lesion distal to the bile duct, while intratumoral ^125^I seed implantation can mainly cover the whole lesion under CT guidance, compensating for the lack of total dose distribution away from the seed strand. Moreover, the brachytherapy of the ^125^I seed strand was effective for about six months, and thereafter, those patients could not receive the re-implantation of fresh ^125^I seed strand to maintain the brachytherapy. In contrast, CT-guided ^125^I seed implantation showed superiority in the replacement of ^125^I seeds when appropriate, thus maintaining long-term brachytherapy to the whole lesion, reducing the lateral pressure of the tumor on the bile duct, delaying the invasion of the bile duct wall, and ensuring the patency of the biliary stent for a certain period. Furthermore, CT scans also can help ensure that the preoperative TPS strategy is precisely performed, resulting in better local control and patient survival.

In the current study, the incidence of complications is consistent with the reported studies [14, 28]. No major complications were founded in the two groups. The majority of complications were manageable with conservative therapy. Patients who received stent placement coupled with seed strand or with CT-guided ^125^I seed implantation had no more complications than stent alone, demonstrating that ^125^I seed combined brachytherapy is safe.

### Limitations

The present research still has several limitations. First, this was a retrospective study with a limited sample size, which reduced the statistical power of the conclusion. Second, indicators like tumor markers were not collected dynamically, therefore the therapy effect on tumor biological behavior was not assessed. Third, this study failed to assess tumor response to ^125^I seed brachytherapy due to the difficulty in evaluating the lesions with the Response evaluation criteria in solid tumors (RECIST) criteria [29].

## Conclusion

In summary, the preliminary findings of this study indicated that the combination of SEMS with ^125^I brachytherapy (including CT-guided intratumoral ^125^I seed implantation and intraluminal^125^I seed insertion) is safe and effective for inoperable MOJ patients. It can improve stent patency and patient survival without increasing the risk of procedure-related complications. In addition, no significant difference was observed between CT-guided ^125^I seed implantation with SEMS and ^125^I seed insertion with SEMS. More large sample prospective studies are required to further confirm our findings.

## Data Availability

The datasets used and/or analyzed during the current study are available from the corresponding author upon reasonable request.

## Abbreviations

SEMS: Self-expandable metallic stents
MOJ: Malignant obstructive jaundice
CT: Computed tomography
TPS: Treatment planning system
MRI: Magnetic resonance imaging
TBIL: Total bilirubin
DBIL: Direct bilirubin
ALT: Alanine aminotransferase
AST: Aspartate aminotransferase
HR: Hazard Ratio
RECIST: Response evaluation criteria in solid tumors
